# Changes in Dietary Intake of Methionine, Folate/Folic Acid and Vitamin B12 and Survival in Postmenopausal Women with Breast Cancer: A Prospective Cohort Study

**DOI:** 10.3390/nu14224747

**Published:** 2022-11-10

**Authors:** Yangbo Sun, Jay H. Fowke, Xiaoyu Liang, Khyobeni Mozhui, Saunak Sen, Wei Bao, Buyun Liu, Linda G. Snetselaar, Robert B. Wallace, Aladdin H. Shadyab, Nazmus Saquib, Ting-Yuan David Cheng, Karen C. Johnson

**Affiliations:** 1 Department of Preventive Medicine, College of Medicine, The University of Tennessee Health Science Center, 66 N. Pauline Street, Memphis, TN 38163, USA; 2 Institute of Public Health, Division of Life Sciences and Medicine, University of Science and Technology of China, Hefei 230026, China; 3 Department of Endocrinology, Division of Life Sciences and Medicine, First Affiliated Hospital of USTC, University of Science and Technology of China, Hefei 230026, China; 4 Department of Epidemiology, College of Public Health, University of Iowa, Iowa City, IA 52242, USA; 5 Herbert Wertheim School of Public Health and Human Longevity Science, University of California, San Diego, CA 92093, USA; 6 College of Medicine, Sulaiman AlRajhi University, Al Bukayriah 51941, Saudi Arabia; 7 College of Public Health and Health Professions, University of Florida, Gainesville, FL 32610, USA

**Keywords:** methionine, postmenopausal breast cancer, cancer survival

## Abstract

Background: Previous experimental studies showed that limiting methionine in the diet of animals or in cell culture media suppresses mammary cancer cell proliferation or metastasis. However, no previous study has investigated the associations of changes in methionine intake with survival among breast cancer survivors. We aimed to examine the association between changes in dietary intake of methionine, folate/folic acid, and vitamin B12 from before to after diagnosis of breast cancer, and mortality among breast cancer survivors. Methods: We included 1553 postmenopausal women from the Women’s Health Initiative who were diagnosed with invasive breast cancer and completed a food frequency questionnaire both before and after breast cancer diagnosis. Multivariable Cox proportional hazards regression models were used to estimate adjusted hazard ratios (HRs) and 95% confidence (CIs) of all-cause and breast cancer mortality associated with changes in methionine intake and changes in folate/folic acid and vitamin B12 intake. Results: Relative to pre-diagnosis, 28% of women decreased methionine intake by ≥20%, 30% of women increased methionine intake by ≥20%, and 42% of women had a relatively stable methionine intake (±19.9%) following breast cancer diagnosis. During a mean 16.1 years of follow up, there were 772 deaths in total, including 195 deaths from breast cancer. Compared to women with relatively stable methionine intake, women with decreased methionine intake had lower risks of all-cause (HR 0.78, 95% CI 0.62–0.97) and breast cancer mortality (HR 0.58, 95% CI 0.37–0.91) in fully adjusted models. In contrast, increased methionine intake or changes in folate/folic acid or vitamin B12 intake were not associated with all-cause or breast cancer mortality. Conclusions: Among breast cancer survivors, decreased methionine intake after breast cancer diagnosis was associated with lower risk of all-cause and breast cancer mortality.

## 1. Introduction

Breast cancer is the most common malignancy in women worldwide [1], accounting for 24.5% of new cancer cases and 15.5% of cancer deaths in 2020. In the US, an estimated 281,550 new cases of invasive breast cancer will be diagnosed, and 43,600 women will die from breast cancer in 2021 [2]. While the overall 5-year relative survival rate is over 90%, survival rates decrease sharply for women diagnosed with advanced stage or metastatic breast cancer [3].

Cancer cells proliferate rapidly compared to normal cells and require larger quantities of glucose and amino acids to sustain this proliferation [4]. Therefore, metabolic regulation in cancer tissue should differ from corresponding healthy tissue [5]. Several prior studies indicate that dietary composition could alter the systemic environment necessary to sustain cell proliferation and cancer progression [6,7]. A well-known yet under-investigated point of focus is the essential amino acid methionine, which is usually obtained from meats, nuts, eggs, and grains, and cannot be produced by human bodies. Aside from serving as a building block for proteins and being essential for normal growth and development, [8] methionine is the precursor to S-adenosylmethionine, which is the principal methyl donor in DNA methylation reactions [9]. Epigenetic processes are now recognized as critical in carcinogenesis and progression across multiple tumor sites, including breast cancer incidence and progression [6,7]. As demand for amino acids increases following malignant transformation, cells may experience a methionine deficiency that could affect these epigenetic processes. Indeed, many experimental studies have shown that limiting methionine in the diet of animals or in cell culture media suppresses mammary cancer cell proliferation or metastasis [10,11,12]. However, to our knowledge, no previous study has investigated the association of change in methionine intake in humans with breast cancer survival.

Breast cancer survivors may be motivated to make lifestyle changes [13], including changes in diet [14], in order to increase survivorship. However, it remains unclear whether changes in methionine intake affects breast cancer survival. Therefore, we aimed to evaluate the prospective long-term associations of dietary methionine intake changes from pre- to post-diagnosis with breast cancer survival using data from the Women’s Health Initiative. Dietary folate acid and vitamin B12 are also analyzed as these nutrients may promote methionine synthesis, to rule out the possibility that the association between methionine and mortality are through these promoters.

## 2. Materials & Methods

### 2.1. Study Participants

The Women’s Health Initiative (WHI) has been previously described in detail [15]. Briefly, between 1993 and 1998, postmenopausal women aged 50 to 79 years at study entry were recruited through 40 clinical centers into either a Clinical Trials (CT) component (*n* = 68,132), or an Observational Study (OS) component (*n* = 93,676 women). The CT included the dietary modification (DM) trial, the hormone therapy (HT) trial, and the calcium and vitamin D (CaD) trial. The CT and OS were closed in 2004–2005 and the participants were invited to continue being followed in the WHI Extension Study since 2005. In the WHI, food frequency questionnaires (FFQs) were evaluated for more than one time only among the DM and OS participants [15]. This study enrolled women in the OS and the comparison group of the DM trial (DM-C). Eligible participants were free of cancer at baseline, diagnosed with invasive breast cancer during follow-up and completed an FFQ pre- and post- diagnosis of invasive breast cancer. We excluded those with invalid dietary data who reported their energy intake <600 or >5000 kcals/day. We further excluded women from DM-C who had also participated in the intervention arm of the HT trial, as it showed that certain menopausal hormone therapy was divergently associated with breast cancer incidence in this population [16]. A total of 7000 women were included in the analysis for pre-diagnosis methionine intake. Among them, 1553 women had dietary information collected again post-diagnosis of breast cancer (shown in Flowchart). Written informed consent was obtained from each subject. Institutional review board approval was obtained from all participating institutions. As this is a secondary data analysis with de-identified data from an existing dataset, the University of Tennessee Health Science Center Institutional Review Board approved that our study is not considered as a study that involves human subjects.

### 2.2. Diet Measurement

A standardized written protocol, centralized training of staff and quality assurance visits by the Clinical Coordinating Center were used to ensure uniform administration of data collection [17]. Dietary information was assessed through a self-administered FFQ developed and validated with characteristics described for the study [18], which were adapted from the Health Habits and Lifestyle Questionnaire [19]. The WHI-FFQ was designed to capture foods relevant for multiethnic and geographically diverse population groups. The WHI FFQ included 122 composite and single food line items asking about frequency of consumption and portion size, 19 adjustment questions related to type of fat intake, and 4 summary questions asking about the usual intake of fruits and vegetables and added fats for comparison with information gathered from the line items. In the DM, all participants completed an FFQ at baseline and year one of follow-up, and one-third of DM participants completed an FFQ each year on a rotating basis thereafter from years two to nine. In the OS, participants completed an FFQ at baseline and at the third year of follow-up. The reliability of the FFQ has been previously assessed. The mean correlation of 30 nutrients estimated by FFQ and eight days of dietary intake from a four-day food record and four 24-h dietary recalls was 0.57. The correlations of energy, percent energy from fat, carbohydrate, and protein estimated from FFQ and four-day food records were 0.37, 0.62, 0.41, and 0.36, respectively [18]. These estimates are similar to estimates in other cohorts [20,21]. Nutrient intake, including methionine intake, was derived from the Nutrition Data Systems for Research [22].

For the current analysis, intake of nutrients was derived from FFQs collected closest to pre- and post-breast cancer diagnosis. Changes in methionine intake pre- and post-cancer diagnosis were classified into three groups (i.e., increased, stable, or decreased) based on a one-half standard deviation in the distribution of changes of methionine intake, which was 20% increased or decreased intake [14,23]. Classifications of changes in folate/folic acid and vitamin B12 intake used a similar approach.

### 2.3. Other Covariates Measurement

Information on demographic characteristics included age, race/ethnicity, socioeconomic status including education and income, and medication use, including use of postmenopausal hormone therapy, and was collected at baseline through self-report. Lifestyle information included smoking status and alcohol intake, and was collected at baseline and annually at years one to nine in the DM, and at baseline and year three in the OS. Recreational moderate–vigorous intensity physical activity, including walking, was assessed by the validated WHI brief physical activity questionnaire, and metabolic equivalent task hours (MET-h)/week of physical activity for each participant was calculated, as described in detail [15,24]. Weight and height were assessed during clinic visits using standard methods at baseline and annually (years 1–11) in the WHI-DM and at baseline and year three in the OS [25]. Body mass index (BMI) was calculated as weight (kg)/height (m)^2^. Lifestyle and BMI changes were assessed, comparing the closest information collected pre- and post-invasive breast cancer diagnosis.

### 2.4. Breast Cancer Outcomes Ascertainment

WHI contacted participants via telephone every six months to determine health status. Self-reported breast cancer diagnosis was followed by acquisition of medical records to verify diagnosis. Reported breast cancer outcomes were adjudicated by centrally trained physician adjudicators who were blinded to treatment assignment at the clinical centers after medical record and pathology report review, and coded according to National Cancer Institute Surveillance, Epidemiology, and End Results guidelines [26]. Breast cancer stages were coded as follows: localized, regional, distant, and unknown. When quantitative immunohistochemical results were available, tumors were also coded as estrogen receptor (ER) status positive or progesterone receptor (PR) status positive if at least 1% of cells stained positive [27].

### 2.5. Death Ascertainment

All medical records were reviewed centrally by the WHI Outcomes Adjudication Committee before assigning the diagnosis in the dataset; similar approaches were used to determine cause of death. Further, vital status of participants was collected through clinical center follow-up of participants and proxies. In addition, periodic searches of the National Death Index were conducted. Causes of death were determined by a review of medical records and death certificate by the Adjudication Committee.

### 2.6. Statistical Analysis

Preliminary analyses compared demographic and other covariates across the three intake groups using analysis of variance for continuous variables and a chi-square test for categorical variables.

We used Cox proportional hazards models to evaluate hazard ratios (HRs) and 95% confidence intervals (CIs) of mortality related to dietary methionine intake changes. We calculated person-years from diagnosis until death, loss to follow-up, or end of the follow-up during the WHI-Extension Study 2 on August 28, 2020. Participants who were alive at study closeout were censored on that closeout date. We constructed multivariable models in several steps. In Model 1, we adjusted for age at diagnosis, race/ethnicity, and pre- and post-diagnosis total energy intake. In Model 2, we additionally adjusted for education, income, OS or DM-C, breast cancer stage, ER status, PR status, postmenopausal hormone therapy use, time from diagnosis to dietary intake measurement after diagnosis, family history of breast cancer, pre- and post-diagnosis smoking status, pre- and post-diagnosis physical activity levels, pre- and post-total protein intake, pre- and post-diagnosis alcohol intake, and mutual adjustment for pre- and post- diagnosis intake of vitamin B12, folate/folic acid, and methionine. Model 3 additionally adjusted for pre- and post-diagnosis BMI, because obesity might be a mediator for the relation between diet and mortality.

We performed stratified analyses according to post-diagnosis smoking status, postmenopausal hormone therapy use, ER status, PR status, cancer stage, post-diagnosis total energy intake reduction, and post-diagnosis obesity status to examine whether the relation between dietary methionine intake changes and survival varied by these variables. We conducted interaction tests via multiplicative interaction terms in the multivariable models. Furthermore, for sensitivity analysis, the main analyses were repeated with women who completed the post-diagnosis FFQ within 6 months of diagnosis excluded, to minimize the possibility of direct effects of treatment on dietary habits. Similarly, we repeated analyses with women who died within 2 years after completion of the post-diagnosis FFQ excluded, to minimize the possibility that the patients’ response was affected by the severe illness. Finally, we repeated the analyses excluding women with stage four breast cancer, or excluding the women in the DM.

All statistical analyses were conducted using SAS (version 9.4) [28]. Statistical significance was set at *p* < 0.05 for all tests being two-sided.

## 3. Results

During an average of 16.1 (standard deviation [SD] 5.6) years of follow-up, there were 772 deaths in total, which included 195 from breast cancer and 577 from causes other than breast cancer. The average time from pre-diagnosis FFQ to breast cancer diagnosis was 1.9 (SD 1.2) years and the average time from diagnosis to post-diagnosis FFQ was 1.5 (SD 1.3) years.

After breast cancer diagnosis, 42% of the participants maintained relatively stable dietary methionine intake (±19.9% change), 28% decreased dietary methionine intake (≥20% decrease), and 30% increased dietary methionine intake (≥20% increase) compared with their pre-diagnosis dietary methionine intake (Table 1). Compared to participants with a relatively stable dietary methionine intake, participants who increased dietary methionine intake had a lower pre-diagnosis intake of total energy, folate/folic and vitamin B12, and had a higher post-diagnosis intake of total energy and total protein, folate/folic, and vitamin B12. They had higher post-diagnosis physical activity levels, were more likely to currently use postmenopausal hormone therapy, and were more obese before diagnosis. On the contrary, those who decreased their dietary methionine intake had a higher pre-diagnosis intake of total energy, folate/folic, and vitamin B12, and had a lower post-diagnosis intake of total energy and total protein, folate/folic, and vitamin B12. They had lower post-diagnosis physical activity levels, were less likely to currently use postmenopausal hormone therapy, and were more obese before diagnosis. The average dietary methionine intake decreased after an invasive breast cancer diagnosis from an average of 1537 (SD 640) mg/day to 1508 (SD 623) mg/day.

Compared with participants with relatively stable dietary methionine intake, participants with decreased dietary methionine intake post-diagnosis had significantly lower risk of all-cause mortality (adjusted HR 0.78, 95% CI 0.62 to 0.97) (Table 2). Similarly, decreased dietary methionine intake post-diagnosis was significantly associated with lower risk of breast cancer mortality (adjusted HR 0.58, 95% CI 0.37 to 0.91) (Table 3). In contrast, increased dietary methionine intake post-diagnosis was not related with risk of mortality from all-cause (adjusted HR 0.97, 95% CI 0.78 to 1.21) or breast cancer (adjusted HR 1.27, 95% CI 0.82 to 1.97). Furthermore, changes in dietary folate/folic acid or vitamin B12 intake were not associated with all-cause or breast cancer mortality after adjustment of other prognostic and dietary factors (Table 2 and Table 3). Higher pre-diagnosis dietary methionine intake was associated with higher risk of all-cause mortality in the second tertile of methionine intake (adjusted HR 1.18, 95% CI 1.01 to 1.39) (Table S1). Higher breast cancer mortality was observed in the highest tertile of methionine intake (adjusted HR 2.61, 95% CI 1.11 to 6.14), as shown in Table S2.

The results were similar when women who completed the post-diagnosis FFQ within six months of diagnosis were excluded, women who died within two years after the collection of the post-diagnosis FFQ were excluded, or women with stage four breast cancer were excluded (Table S3). The association was not statistically significant when the analysis was repeated among women from OS, probably due to reduced sample size. The associations did not differ by post-diagnosis smoking status, use of postmenopausal hormone therapy, ER status, PR status, cancer stage, post-diagnosis total energy intake reduction, or post-diagnosis obesity status (*p* values ≥ 0.07).

## 4. Discussion

This large prospective cohort study has shown that a decrease in dietary methionine intake post-diagnosis of invasive breast cancer was associated with lower risk of mortality from all-cause and breast cancer. However, increased dietary methionine intake was not associated with survival among breast cancer survivors. Dietary intake of folate/folic acid or vitamin B12 was not associated with breast cancer survival.

To our knowledge, our study is the first to evaluate the relation between changes in methionine intake and breast cancer survival in humans. The findings are biologically plausible. Methionine is an essential amino acid derived from the diet. Aside from serving as a building block for proteins, methionine is also imbedded in the pathways pivotal for DNA methylation and polyamine synthesis [9]. In breast tumor cell cultures, limiting methionine in the cell culture media limits cell proliferation [29]. In animal models, a methionine-deficient diet suppresses mammary tumor metastasis [10,11,12]. Possible mechanisms of antitumor effects of methionine restriction include the pervasive dependence on exogenous methionine in cancer due to a defect in methionine synthesis, and due to contextual factors that shape individual tumors [10,11,12,30]. The precursor homocysteine is metabolized either through the methionine cycle to produce methionine or through the transsulfuration pathway to synthesize cysteine [31]. Most methionine-dependent breast cancer cell lines harbor a *PIK3CA* genomic mutation and decreased expression of *SLC7A11*, which is a gene that encodes a cystine transporter also known as xCT that is correlated with increased methionine dependency in breast cancer cells [31]. Concurrent with decreased cystine uptake through xCT, oncogenic *PIK3CA* mutant cells use homocysteine through the transsulfuration pathway to synthesize cysteine. Consequently, less homocysteine is available to produce methionine, thus contributing to methionine dependency [31].

Although no previous study has examined changes in diet methionine intake in relation to breast cancer survival in humans, several previous studies examining the association between methionine and breast cancer risk have shown inconsistent findings. A meta-analysis of seven prospective and six case-control studies found that premorbid high vs. low methionine intake was significantly associated with breast cancer risk among postmenopausal women but not among pre-menopausal women [32]. A separate meta-analysis focused on the methionine synthase (MTR) gene polymorphism A2756G (rs1805087), a common polymorphism thought to possibly lower MTR activity leading to lower methionine, suggested that there is no significant association between A2756G and breast cancer risk [33].

## 5. Strengths and Limitations

Strengths of this study include the large sample size, prospective design, and long-term follow-up. In WHI, there is also detailed data on covariates that potentially cofound the associations between changes in dietary methionine intake and survival; thus, the role of methionine in mortality after diagnosis of breast cancer was examined thoroughly. This study also has limitations. First, dietary assessment is susceptible to measurement error. Second, a change in dietary methionine intake may not lead to a similar change in systemic methionine levels, as there is often a lag between the changes in dietary methionine intake and the change in circulating methionine concentrations. However, this study included FFQ collection on average 1.5 years after diagnosis, such that any temporary adaptations may be minimized. Third, it is possible that cancer survivors with a poorer prognosis would be more likely to change their diet post diagnosis. However, we excluded participants who had cancer at baseline, controlled for breast cancer stage, ER status, and PR status, and excluded persons who died within 2 years of collection of the post-diagnosis FFQ. Fourth, cancer treatment information was not available. However, we controlled for cancer stage, which is correlated with treatment in survivors in other studies of breast cancer survivors [34], although it is not a perfect proxy for treatment. Fifth, WHI recruited postmenopausal women aged 50–79 at study entry, thus, the results may not be generalized to younger breast cancer survivors or women diagnosed with premenopausal breast cancer. Sixth, as an observational study, residual confounding is possible. However, we have controlled for a variety of potential confounders for the association between methionine intake and mortality, and no substantial differences were shown in the results. Finally, diet was assessed only once pre- and post-diagnosis of breast cancer and may have changed over time.

These findings provide evidence for possible development of low-methionine therapeutic diet or methionine lowering drugs by depleting methionine from cancer cells to enhance response to breast cancer chemotherapy or radiation therapy [12,29,35]. Furthermore, our results suggest that the benefit of methionine reduction may generalize across tumor stage, although this needs confirmation in future studies in order to determine which patients may benefit most from methionine interventions.

In conclusion, among postmenopausal invasive breast cancer survivors, decrease in dietary methionine intake after diagnosis of breast cancer was associated with lower risk of all-cause and breast cancer mortality. Further studies, including both observational studies and clinical trials, are needed to fully understand how dietary methionine intake changes after breast cancer diagnosis affect long-term health among breast cancer survivors. Lastly, studies exploring the underlying mechanisms are needed.

## Figures and Tables

**Table 1:** Characteristics according to dietary methionine intake changes from before to after diagnosis of invasive breast cancer among 1553 breast cancer survivors.

	Change in Dietary Methionine Intake			
	Increase (≥20%)	No Change or Stable (±19.9%)	Decrease (≥20%)	*p* Vales
Number of participants	454	658	441	
Age at diagnosis, years	65.6 (7.1)	66.1 (6.9)	66.3 (6.5)	0.29
Race/Ethnicity, n (%)				0.19
Non-Hispanic white	399 (87.9)	591(89.8)	391 (88.7)	
Nom-Hispanic black	24 (5.3)	37 (5.6)	22 (5.0)	
Hispanic	13 (2.9)	10 (1.5)	16 (3.6)	
Other (American Indian or Alaskan Native, Asian or Pacific Islander and others)	18 (4.0)	17 (2.6)	12 (2.7)	
Unknown	0 (0.0)	3 (0.5)	0 (0.0)	
Pre-diagnosis total energy intake, kcal/day	1367 (470)	1622 (563)	1825 (677)	<0.001
Post-diagnosis total energy intake, kcal/day	1768 (594)	1564 (529)	1297 (470)	<0.001
Education, n (%)				0.08
High school or less	98 (21.6)	151 (23.0)	128 (29.0)	
Some college	126 (27.8)	175 (26.6)	123 (27.9)	
College degree	74 (16.3)	86 (13.1)	48 (10.9)	
Postgraduate	153 (33.7)	239 (36.3)	140 (31.8)	
Missing	3 (0.7)	7 (1.1)	2 (0.5)	
Annual income, n (%)				0.63
<$20,000	48 (10.6)	82 (12.5)	51 (11.6)	
$20,000–49,999	190 (41.9)	278 (42.3)	188 (42.6)	
>$50,000	191 (42.1)	268 (40.7)	171 (38.8)	
Missing	25 (5.5)	30 (4.6)	31 (7.0)	
WHI component/arm, n (%)				0.35
Observational study	273 (60.1)	397 (60.3)	248 (56.2)	
DM-control	181 (39.9)	261 (39.7)	193 (43.8)	
Stage of breast cancer, n (%)				0.17
Localized	348 (76.7)	495 (75.2)	312 (70.8)	
Regional	100 (22.0)	149 (22.6)	122 (27.7)	
Distant	4 (0.9)	4 (0.6)	4 (0.9)	
Unknown	2 (0.4)	10 (1.5)	3 (0.7)	
Estrogen receptor status, n (%)				0.87
Positive	343 (75.6)	497 (75.5)	324 (73.5)	
Negative	61 (13.4)	95 (14.4)	69 (15.7)	
Unknown	50 (11.0)	66 (10.0)	48 (10.9)	
Progesterone receptor status, %				0.68
Positive	286 (63.0)	417 (63.4)	263 (59.6)	
Negative	112 (24.0)	157 (23.9)	122 (27.7)	
Unknown	56 (12.3)	84 (12.8)	56 (12.7)	
Postmenopausal hormone therapy, n (%)				0.04
Never	135 (29.7)	200 (30.4)	141 (32.0)	
Past	48 (10.6)	67 (10.2)	68 (15.4)	
Current	271 (59.7)	391 (59.4)	232 (52.6)	
Time from diagnosis to post-diagnosis FFQ assessment, years				0.26
Had female relatives that had breast cancer, n (%)				0.36
No	128 (28.2)	167 (25.4)	117 (26.5)	
Yes	101 (22.3)	176 (26.8)	99 (22.5)	
Unknown	225 (49.6)	315 (47.9)	225 (51.0)	
Pre-diagnosis smoking status, n (%)				0.09
Non-current smoker	424 (93.4)	629 (95.6)	414 (93.9)	
Current smoker	29 (6.4)	25 (3.8)	21 (4.8)	
Missing	1 (0.2)	4 (0.6)	6 (1.4)	
Post-diagnosis smoking status, n (%)				0.77
Non-current smoker	421 (92.8)	613 (93.2)	414 (93.9)	
Current smoker	19 (4.2)	21 (3.2)	12 (2.7)	
Missing	14 (3.1)	24 (3.7)	15 (3.4)	
Pre-diagnosis physical activity levels, MET-hours/week, n (%)				0.52
<10	223 (49.1)	318 (48.3)	235 (53.3)	
≥10	215 (47.4)	320 (48.6)	191 (43.3)	
Missing	16 (3.5)	20 (3.0)	15 (3.4)	
Post-diagnosis physical activity levels, MET-hours/week, n (%)				0.10
<10	227 (50.0)	343 (52.1)	258 (58.5)	
≥10	213 (46.9)	290 (44.1)	169 (38.3)	
Missing	14 (3.1)	25 (3.8)	14 (3.2)	
Pre-diagnosis alcohol intake, g/day	5.9 (12.0)	6.2 (11.6)	6.3 (12.7)	0.29
Post-diagnosis alcohol intake, g/day	5.3 (10.1)	5.5 (11.7)	4.1 (9.3)	0.08
Pre-diagnosis folate/folic acid intake, mcg DFE/day	299 (170)	336 (194)	351 (194)	<0.001
Post-diagnosis folate/folic acid intake, mcg DFE/day	507 (203)	471 (193)	391 (165)	<0.001
Pre-diagnosis vitamin B12 intake, mcg/day	4.8 (2.7)	6.1 (3.2)	6.9 (3.6)	<0.001
Post-diagnosis vitamin B12 intake, mcg/day	7.2 (3.9)	6.0 (3.2)	4.7 (2.4)	<0.001
Pre-diagnosis total protein intake, g/day	54.6 (19.7)	68.0 (23.7)	80.0 (31.3)	<0.001
Post-diagnosis total protein intake, g/day	79.9 (28.4)	66.7 (22.7)	51.7 (19.8)	<0.001
Pre-diagnosis BMI status, n (%)				0.01
≤24.9	173 (38.1)	252 (38.3)	133 (30.2)	
25.0–29.9	151 (33.3)	219 (33.3)	145 (32.9)	
≥ 30.0	125 (27.5)	185 (28.1)	161 (36.5)	
Missing	5 (1.1)	2 (0.3)	2 (0.5)	
Post-diagnosis BMI status, n (%)				0.56
≤24.9	145 (31.9)	234 (35.6)	141 (32.0)	
25.0–29.9	145 (31.9)	213 (32.4)	136 (30.8)	
≥30.0	119 (26.2)	157 (23.9)	126 (28.6)	
Missing	45 (9.9)	54 (8.2)	38 (8.6)	

**Table 2:** Changes in dietary intake of methionine, folate/folic acid, and vitamin B12 pre- and post-diagnosis of invasive breast cancer in relation to all-cause mortality among 1553 breast cancer survivors.

	Change in Dietary Methionine Intake		
	Increase (≥20%)	No Change or Stable (±19.9%)	Decrease (≥20%)
NO. of deaths/participants	209/454	341/658	222/441
Model 1 ^a^	1.00 (0.82, 1.21)	ref	0.88 (0.73 to 1.06)
	*p* = 0.97		*p* = 0.18
Model 2 ^b^	1.00 (0.80, 1.25)	ref	0.79 (0.64 to 0.98)
	*p* = 0.99		*p* = 0.03
Model 3 ^c^	0.97 (0.78, 1.21)	ref	0.78 (0.62 to 0.97)
	*p* = 0.78		*p* = 0.02
	Change in dietary folate/folic acid intake		
	Increase (≥20%)	No change or stable (±19.9%)	Decrease (≥20%)
NO. of deaths/participants	484/986	179/363	109/204
Model 1 ^a^	0.93 (0.78, 1.11)	ref	1.10 (0.86 to 1.40)
	*p* = 0.41		*p* = 0.47
Model 2 ^b^	0.94 (0.78, 1.14)	ref	1.05 (0.82 to 1.36)
	*p* = 0.53		*p* = 0.69
Model 3 ^c^	0.97 (0.80, 1.17)	ref	1.02 (0.79 to 1.32)
	*p* = 0.74		*p* = 0.85
	Change in dietary vitamin B12 intake		
	Increase (≥20%)	No change or stable (±19.9%)	Decrease (≥20%)
NO. of deaths/participants	254/537	259/528	259/488
Model 1 ^a^	1.11 (0.93, 1.33)	ref	1.09 (0.91 to 1.30)
	*p* = 0.25		*p* = 0.37
Model 2 ^b^	1.11 (0.92, 1.34)	ref	1.04 (0.85 to 1.26)
	*p* = 0.30		*p* = 0.72
Model 3 ^c^	1.11 (0.91, 1.34)	ref	1.01 (0.83 to 1.23)
	*p* = 0.30		*p* = 0.89

^a^ Model 1: Adjusted for age at diagnosis, race or ethnicity, and pre- and post-diagnosis total energy intake. ^b^ Model 2: Adjusted for covariates included in model 1 plus education, income, observational study vs. dietary modification clinical trial comparison arm, breast cancer stage, estrogen receptor status, progesterone receptor status, use of postmenopausal hormone therapy, time from diagnosis to post-diagnosis dietary intake assessment, family history of breast cancer, pre- and post-diagnosis smoking status, pre- and post-diagnosis physical activity levels, pre- and post-diagnosis total protein intake, pre- and post-diagnosis alcohol intake, and mutual adjustment for pre- and post- diagnosis vitamin B12 intake, pre- and post- diagnosis folate/folic acid intake, and pre- and post- diagnosis methionine intake. ^c^ Model 3: Adjusted for covariates included in model 2 plus pre- and post-diagnosis BMI.

**Table 3:** Changes in dietary intake of methionine, folate/folic acid, and vitamin B12 intake pre- and post-diagnosis of invasive breast cancer in relation to breast cancer mortality among 1553 breast cancer survivors.

	Change in Dietary Methionine Intake		
	Increase (≥20%)	No Change or Stable (±19.9%)	Decrease (≥20%)
NO. of deaths/participants	60/454	84/658	51/441
Model 1 ^a^	1.21 (0.84, 1.75)	ref	0.76 (0.51 to 1.12)
	*p* = 0.32		*p* = 0.17
Model 2 ^b^	1.31 (0.85, 2.03)	ref	0.58 (0.37 to 0.91)
	*p* = 0.22		*p* = 0.02
Model 3 ^c^	1.27 (0.82, 1.97)	ref	0.58 (0.37 to 0.91)
	*p* = 0.29		*p* = 0.02
	Change in dietary folate/folic acid intake		
	Increase (≥20%)	No change or stable (±19.9%)	Decrease (≥20%)
NO. of deaths/participants	129/986	39/363	27/204
Model 1 ^a^	1.20 (0.83, 1.72)	ref	1.21 (0.73 to 2.00)
	*p* = 0.34		*p* = 0.45
Model 2 ^b^	1.13 (0.77, 1.67)	ref	1.06 (0.62 to 1.82)
	*p* = 0.52		*p* = 0.83
Model 3 ^c^	1.17 (0.80, 1.73)	ref	1.08 (0.63 to 1.85)
	*p* = 0.42		*p* = 0.79
	Change in dietary vitamin B12 intake		
	Increase (≥20%)	No change or stable (±19.9%)	Decrease (≥20%)
NO. of deaths/participants	67/537	69/528	59/488
Model 1 ^a^	1.01 (0.71, 1.43)	ref	0.88 (0.61 to 1.28)
	*p* = 0.96		*p* = 0.51
Model 2 ^b^	1.03 (0.71, 1.50)	ref	0.93 (0.64 to 1.37)
	*p* = 0.85		*p* = 0.58
Model 3 ^c^	1.01 (0.69, 1.47)	ref	0.89 (0.60 to 1.31)
	*p* = 0.76		*p* = 0.43

^a^ Model 1: Adjusted for age at diagnosis, race or ethnicity, and pre- and post-diagnosis total energy intake. ^b^ Model 2: Adjusted for covariates included in model 1 plus education, income, observational study vs. dietary modification clinical trial comparison arm, breast cancer stage, estrogen receptor status, progesterone receptor status, use of postmenopausal hormone therapy, time from diagnosis to post-diagnosis dietary intake assessment, family history of breast cancer, pre- and post-diagnosis smoking status, pre- and post-diagnosis physical activity levels, pre- and post-diagnosis total protein intake, pre- and post-diagnosis alcohol intake, and mutual adjustment for pre- and post- diagnosis vitamin B12 intake, pre- and post- diagnosis folate/folic acid intake, and pre- and post- diagnosis methionine intake. ^c^ Model 3: Adjusted for covariates included in model 2 plus pre- and post-diagnosis BMI.

## Data Availability

The data underlying this article were provided by the Women’s Health Initiative by permission. Data will be shared on request to the corresponding author with permission of the Women’s Health Initiative.

## Supplementary Materials

The following supporting information can be downloaded at: https://www.mdpi.com/article/10.3390/nu14224747/s1, Table S1: Pre-diagnosis dietary methionine intake in relation to mortality among 7000 women from the Women’s Health Initiative; Table S2: Post-diagnosis dietary methionine intake in relation to mortality among 1553 women from the Women’s Health Initiative; Table S3: Sensitivity analysis of the association between changes in dietary methionine intake and mortality.
