# Supplementary material for: Changes in Dietary Intake of Methionine, Folate/Folic Acid and Vitamin B12 and Survival in Postmenopausal Women with Breast Cancer: A Prospective Cohort Study
Source: Nutrients. 2022 Nov 10;14(22):4747. doi: 10.3390/nu14224747 (PMC9699275; doi:10.3390/nu14224747)
Supplement: Supplementary file 1 [file nutrients-14-04747-s001.zip › nutrients-1958779-supplementary.pdf]

Supplementary Table S1 Pre-diagnosis dietary methionine intake in relation to mortality among 7000 women from the Women's Health Initiative

	Pre-diagnosis dietary methionine intake (g/day)			
	Tertile 1 (<1.22)	Tertile 2 (1.23-1.69)	Tertile 3 (>1.70)	*P* for trend
All-cause mortality				
NO. of deaths/participants	938/2325	926/2338	864/2337	
Model 1	ref	0.96(0.87, 1.06)	0.92(0.80, 1.05)	0.05
Model 2	ref	1.20(1.03, 1.41)	1.23(0.99, 1.55)	0.99
Model 3	ref	1.18(1.01, 1.39)	1.19(0.95, 1.49)	0.90
Breast cancer mortality				
NO. of deaths/participants	233/2325	238/2338	208/2337	
Model 1	ref	0.96(0.79, 1.18)	0.77(0.59, 1.01)	0.24
Model 2	ref	1.28(0.92, 1.77)	1.06(0.67, 1.68)	0.71
Model 3	ref	1.25(0.91, 1.73)	1.04(0.65, 1.64)	0.66

Model 1: Adjusted for age at diagnosis, race or ethnicity, and pre-diagnosis total energy intake.
Model 2: Adjusted for covariates included in model 1 plus education, income, observational study vs. dietary modification clinical trial comparison arm, breast cancer stage, estrogen receptor status, progesterone receptor status, use of postmenopausal hormone therapy, family history of breast cancer, pre-diagnosis smoking status, pre-diagnosis physical activity levels, pre-diagnosis total protein intake, pre-diagnosis alcohol intake, pre-diagnosis vitamin B12 intake, and pre-diagnosis folate/folic acid intake.
Model 3: Adjusted for covariates included in model 2 plus pre-diagnosis BMI.

Supplementary Table S2 Post-diagnosis dietary methionine intake in relation to mortality among 1553 women from the Women's Health Initiative

	Post-diagnosis dietary methionine intake (g/day)			
	Tertile 1 (<1.19)	Tertile 2 (1.20-1.69)	Tertile 3 (>1.70)	*P* for trend
All-cause mortality				
NO. of deaths/participants	273/517	252/518	247/518	
Model 1	Ref	0.92(0.76, 1.12)	0.90(0.69, 1.18)	0.47
Model 2	ref	1.01(0.75, 1.35)	1.13(0.74, 1.72)	0.02
Model 3	ref	1.02(0.76, 1.37)	1.10(0.72, 1.68)	0.03
Breast cancer mortality				
NO. of deaths/participants	64/517	64/518	67/518	
Model 1	Ref	1.05(0.71, 1.56)	1.18(0.69, 2.01)	0.26
Model 2	ref	1.40(0.77, 2.56)	2.77(1.18, 6.52)	0.04
Model 3	ref	1.37(0.75, 2.51)	2.61(1.11, 6.14)	0.07

Model 1: Adjusted for age at diagnosis, race or ethnicity, pre- and post-diagnosis total energy intake, and pre-diagnosis methionine intake.
Model 2: Adjusted for covariates included in model 1 plus education, income, observational study vs. dietary modification clinical trial comparison arm, breast cancer stage, estrogen receptor status, progesterone receptor status, use of postmenopausal hormone therapy, time from diagnosis to post-diagnosis dietary intake assessment, family history of breast cancer, pre- and post-diagnosis smoking status, pre- and post-diagnosis physical activity levels, pre- and post-diagnosis total protein intake, pre- and post-diagnosis alcohol intake, pre- and post-diagnosis vitamin B12 intake, and pre- and post- diagnosis folate/folic acid intake,.
Model 3: Adjusted for covariates included in model 2 plus pre- and post-diagnosis BMI.

Supplementary Table S3 Sensitivity analysis of the association between changes in dietary methionine intake and mortality

	Excluding women who returned the postdiagnosis FFQ within 6 months of diagnosis		
	Increase (≥20%)	No change or stable (±19.9%)	Decrease (≥20%)
NO. of deaths/participants	173/376	282/536	180/353
All-cause mortality	0.94(0.74, 1.21)	ref	0.78(0.61, 0.99)
NO. of deaths/participants	49/376	70/536	40/353
Breast cancer mortality	1.24(0.76, 2.00)	ref	0.54(0.32, 0.92)
	Excluding women who died within 2 years after completion of the post-diagnosis FFQ		
	Increase (≥20%)	No change or stable (±19.9%)	Decrease (≥20%)
NO. of deaths/participants	196/441	324/641	208/427
All-cause mortality	0.97(0.77, 1.22)	ref	0.77(0.61, 0.96)
NO. of deaths/participants	55/441	74/641	41/427
Breast cancer mortality	1.37(0.86, 2.18)	ref	0.53(0.32, 0.87)
	Excluding women with stage 4 breast cancer		
	Increase (≥20%)	No change or stable (±19.9%)	Decrease (≥20%)
NO. of deaths/participants	206/450	337/654	218/437
All-cause mortality	0.98(0.78, 1.22)	ref	0.77(0.62, 0.96)
NO. of deaths/participants	57/450	81/654	48/437
Breast cancer mortality	1.26(0.81, 1.96)	ref	0.56(0.35, 0.89)
	Including only women in the OS		
	Increase (≥20%)	No change or stable (±19.9%)	Decrease (≥20%)
NO. of deaths/participants	129/273	204/397	125/248
All-cause mortality	0.96(0.71, 1.28)	ref	0.95(0.71, 1.26)
NO. of deaths/participants	43/273	52/397	32/248
Breast cancer mortality	1.48(0.85, 2.56)	ref	0.69(0.38, 1.24)

Model 1: Adjusted for age at diagnosis, race or ethnicity, and pre-diagnosis total energy intake.

Model 2: Adjusted for covariates included in model 1 plus education, income, observational study vs. dietary modification clinical trial comparison arm, breast cancer stage, estrogen receptor status, progesterone receptor status, use of postmenopausal hormone therapy, family history of breast cancer, pre-diagnosis smoking status, pre-diagnosis physical activity levels, pre-diagnosis total protein intake, pre-diagnosis alcohol intake, pre-diagnosis vitamin B12 intake, and pre-diagnosis folate/folic acid intake.

Model 3: Adjusted for covariates included in model 2 plus pre-diagnosis BMI.
